# Supplementary material for: Quasi-one-dimensional hydrogen bonding in nanoconfined ice
Source: Nat Commun. 2024 Aug 24;15:7301. doi: 10.1038/s41467-024-51124-z (PMC11344787; doi:10.1038/s41467-024-51124-z)
Supplement: Supplementary file 1 — Supplementary Information [file 41467_2024_51124_MOESM1_ESM.pdf]

Supplementary Information:
Quasi-one-dimensional hydrogen bonding in nanoconfined ice

Pavan Ravindra,[1,2,*] Xavier R. Advincula,[1,3,4,*] Christoph Schran,[3,4]
Angelos Michaelides,[1,4,†] and Venkat Kapil[1,4,5,6,‡]

[1] *Yusuf Hamied Department of Chemistry, University of Cambridge, Lensfield Road, Cambridge, CB2 1EW, UK*
[2] *Department of Chemistry, Columbia University, 3000 Broadway, New York, NY 10027, USA*
[3] *Cavendish Laboratory, Department of Physics, University of Cambridge, Cambridge, CB3 0HE, UK*
[4] *Lennard-Jones Centre, University of Cambridge, Trinity Ln, Cambridge, CB2 1TN, UK*
[5] *Department of Physics and Astronomy, University College London, 17-19 Gordon St, London WC1H 0AH, UK*
[6] *Thomas Young Centre and London Centre for Nanotechnology, 19 Gordon St, London WC1H 0AH, UK*

Supplementary Note I. Computational Details

A. Simulation model of nanoconfined water

The system considered in this work is a set of water molecules trapped between two parallel confining sheets, as shown in Supplementary Figure 1. The two parallel confining sheets are kept 5.0 Å apart, measured as the distance between the centers of the atomic nuclei in each confining sheet. This results in the water molecules forming a single monolayer parallel to each confining sheet.

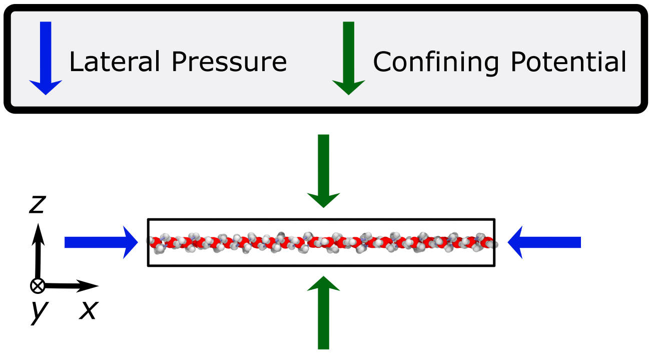

Supplementary Figure 1. | **Simulation setup overview.** Schematic overview of the confined system and the directions of the lateral pressure and confining potential.

The total potential energy of this system is written as a sum of the potential energy of the water molecules and a confining potential [1–8]. The confining potential is a Morse potential fitted to Quantum Monte Carlo water-carbon total energies used in Refs. 4, 8, 9. The confining potential is uniform in the plane of confinement and depends explicitly on the perpendicular distance of a water molecule from the confining sheets. The total potential energy of the water molecules is represented using a high-dimensional Behler-Parrinello neural network potential [10] fitted to total energies and forces calculated at revPBE0-D3 density functional theory level [11, 12].

B. Machine Learning Potential

To train the machine learning potential, we employ an active learning protocol called query by committee (QbC) implemented by Schran et al. [13]. In the QbC approach, the MLP committee is initially trained on a small set of structures. Subsequent structures are added to the training set from a larger dictionary only if they have large committee disagreements. By only adding structures with large committee disagreements to our training set, we can iteratively and systematically build up a compact training set of informative structures. Previous work used the bulk and interfacial water potential from Ref. 13 as a starting point and supplemented additional monolayer and bulk structures at a variety of temperatures and lateral pressures [8]. In this work, we further added monolayer, bilayer, trilayer, and tetralayer structures from simulations of confining widths of 6.5, 9.0, 9.5, and 11.5 Å. We also included structures from PIMD simulation trajectories so that the training dataset includes the new configurations that are accessible with the inclusion of nuclear quantum effects. The average training error of the resulting MLP used in this work is 83.82 meV/Å for forces and 3.38 meV/water for energies.

To assess the neural network's ability to generalize to unseen configurations, we compared the energies and forces produced by the MLP to those produced by the reference revPBE0-D3 functional on new structures that were not seen during training time. These structures were generated by running 2 nanosecond $NP_{xy}T$ simulations at 2.0 GPa starting from each of the solid phases identified in Ref. 8. Each solid phase was simulated at 100 K, 300 K, and 600 K. For some of these simulations, the solid phase was metastable, and so the trajectory remained in the solid phase. For other simulations, the solid phase was unstable, and the water molecules in the simulation entered a disordered phase or glassy phase (depending on the conditions). Hence,

* These authors contributed equally.
† am452@cam.ac.uk
‡ vk380@cam.ac.uk

Supplementary Table I. | **Force field validation.** Average MLP errors on configurations that were not seen during training time. These configurations comes from $NP_{xy}T$ simulations at 2.0 GPa covering a wide range of newly-generated ordered and disordered structures.

Temperature	Energy MAE [meV/water]	Avg Atomic Force RMSE [meV/Å]
100 K	5.8	67.6
300 K	4.8	81.8
600 K	5.6	103.4

these trajectories contained a diverse set of ordered and disordered configurations that span a broad range of local molecular configurations. We computed the errors between the MLP's predictions and the reference revPBE0-D3 calculations over all of the configurations generated in each of these trajectories. The resulting mean absolute errors (MAE) for the energies and root-mean-squared errors (RMSE) for average atomic forces are shown in Supplementary Table I. These errors are similar in magnitude to the errors of MLPs employed in other studies [8, 14], which have been validated against reference DFT simulations. We also note that this model [15] (and its predecessors based on the same machine learning architecture [16, 17]) have been validated against experimental IR, Raman and sum frequency generation spectra, demonstrating an excellent description of real-time dynamics of aqueous systems.

C. vdW pressure

An important component of this system is the vdW pressure (or lateral pressure) experienced between the sheets of the confining material. For instance, if the confining material is graphene, the attractive vdW forces between the two graphene sheets will pull them closer together. If a pocket of water is encapsulated between these two sheets, the graphene sheets will begin to close inwards, pushing the water pocket into an increasingly small region of space. This will continue until the internal pressure of the water balances this attractive vdW force. This results in an effective lateral pressure from the termination of the pocket that acts in the directions perpendicular to the confining potential [18]. We model this lateral pressure by running simulations in the $NP_{xy}T$ ensemble.

D. Molecular dynamics simulations

The molecular dynamics simulations were performed using `i-PI` [19] with the `n2p2` [20] code interfaced with `LAMMPS` [21] to perform MLP energy and gradient calculations. Molecular dynamics simulations used cells containing 144 and 576 molecules. To sample the $NP_{xy}T$ ensemble, we employed a flexible barostat [22] (constrained to only allow lateral cell fluctuations) with a time constant of 1000 fs and optimally-damped generalized Langevin equation thermostats [23] to control the temperature of the physical and the barostat degrees of freedom. To calculate dynamical properties, we performed simulations in the NVT ensemble using a stochastic velocity rescaling thermostat [24] with a time constant of 100 fs. Using a BAOAB splitting of the isothermal isobaric Liouville operator [25, 26], we used a timestep of 1.0 fs to run simulations for 1 ns.

E. Confining Potential

As discussed earlier, we used an implicit confining potential to emulate the interactions between the water molecules and the confining material. The two parallel walls are kept at a fixed distance of 5 Å away from each other, and the nanoconfined water molecules all lie between these two implicit walls. If a water molecule is a distance z away from one of the walls, the potential that it experiences is a Morse potential of the form:

$$V_{morse} = D_0\left[\left(1 - e^{-a(z-z_0)}\right)^2 - 1\right] \tag{1}$$

with the parameter values $D_0 = 5.78 \times 10^{-2}$ eV, $z_0 = 3.85$ Å, and $a = 0.92$ Å^{-1}. These values were obtained from a fit to water-carbon Quantum Monte Carlo (QMC) interaction energies [27].

F. Path integral molecular dynamics simulations

The path integral molecular dynamics (PIMD) simulations used the same software and parameters as the molecular dynamics simulations. We employed simulation cells containing 144 molecules with 32 imaginary time slices. We used an optimally-damped generalized Langevin equation thermostat to control the barostat temperature and a local path-integral Langevin equation thermostat [28] to thermalize the physical degrees of freedom. Using a BAOAB splitting of the path-integral isothermal isobaric Liouville operator, we used a timestep of 0.5 fs to run simulations for 1 ns. For structural and thermodynamic quantities (e.g., free energy profiles), we average results over all the imaginary time slices from the PIMD simulations. For dynamical quantities, we use the centroids of the imaginary time slices.

G. Computing free energy profiles

We used a simple binning procedure to compute free energy profiles along a particular variable, x. Given a

molecular dynamics trajectory, we assemble a list of all values that the variable x takes on in each frame of the trajectory. We then compute the probability histogram $P(x)$ with an appropriately chosen number of bins. The free energy profile in units of $k_B T$ is then reported as $F(x) = -\log(P(x))$, where k_B is Boltzmann's constant. To account for quantum nuclear effects, we perform the binning procedure on the trajectory of the individual imaginary time slices of our PIMD simulations and average the histograms.

Supplementary Note II. Hydrogen Bond Definition

We employ the geometric hydrogen bond definition of Luzar and Chandler [29]. Two water molecules are considered to be (putatively) hydrogen-bonded if they satisfy the following two conditions: (1) The distance between the two oxygen atoms is less than 3.5 Å, and (2) the angle formed by the O-O vector and one of the O-H bond vectors is less than 30°. The sensitivity of the hydrogen bonding trends for the flat-rhombic phase with respect to the parameters of this geometric definition are shown in Supplementary Figure 2.

In Fig. 4(a) of the main text, we showed that employing such a geometric hydrogen bond definition resulted in the number of hydrogen bonds seeming to increase as temperature is raised. This contradicts the expected behavior from a standard energy-entropy trade-off perspective. We suggested that this was because this geometric definition still included fleeting hydrogen bonds that only lasted for times shorter than intermolecular timescales. Employing the definition by Schienbein and Marx [30], we also computed the number of dynamical hydrogen bonds as a function of temperature. Here, a dynamical hydrogen bond is one that satisfies the above geometric criteria for at least 0.2 ps, the typical timescale for intermolecular oscillations for monolayer ice as well as supercritical and room temperature water [30]. The resulting dynamical hydrogen bond count exhibits the expected decreasing trend, as shown in Supplementary Figure 3.

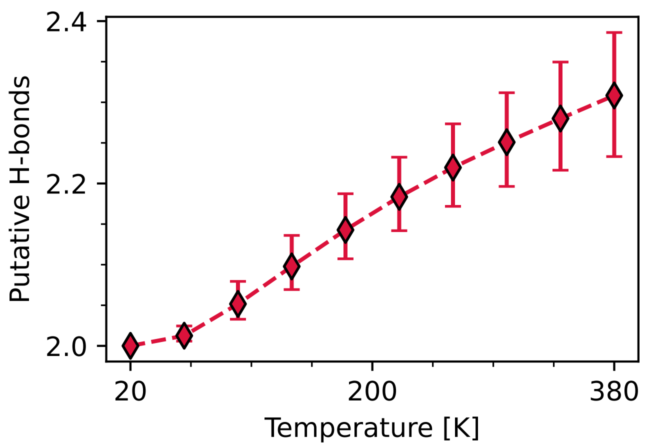

Supplementary Figure 2. | Geometric hydrogen bond definition. The number of (putative) hydrogen bonds in our classical simulations of the flat-rhombic phase as a function of temperature. The main curve is the same as in the classical (red) curve of Fig. 4(a) in the main text. The upper/lower error bounds are obtained by increasing/decreasing the two hydrogen bonding criteria in the text by 5% each.

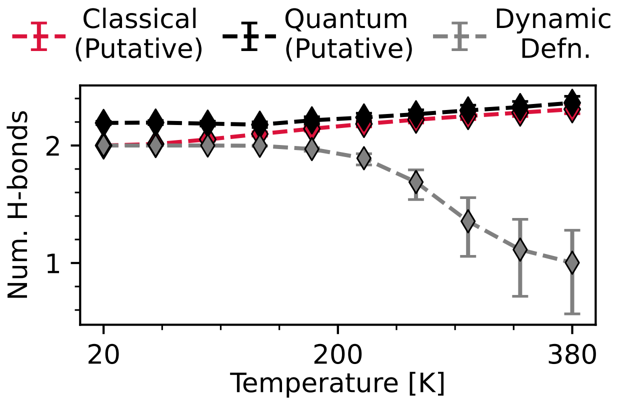

Supplementary Figure 3. | Dynamical hydrogen bond count. The grey data points show the number of hydrogen bonds that survive at least 0.2 picoseconds, with the lower and upper error bounds given by a 0.3 and 0.1 picosecond cutoff, respectively. The red and black lines are the same as in Fig. 4(a) of the main text, showing the number of geometric/putative hydrogen bonds as a function of temperature. The error bars for the putative lines show the standard error of the mean, as computed by block averaging over 10 blocks.

Supplementary Note III. σ Order Parameter Definition

To describe the concerted motion shown in Fig. 6(a) of the main text, we define a σ order parameter to distinguish the two states. The side views of Fig. 6(a) show that the individual dipole directions of each row of water molecules change when the flat-rhombic phase exchanges between these states. We use ϕ_{ij} to denote the ϕ angle of the water molecule in row i and column j of the flat-rhombic lattice. We define the switching parameter b_i that alternates signs between rows and the σ order parameter as

$$b_i = \begin{cases} +1 & \text{odd } i \\ -1 & \text{even } i \end{cases} \tag{2}$$

$$\sigma = \frac{1}{n} \sum_{i,j} b_i \phi_{ij}, \tag{3}$$

where n is the number of water molecules in our system. The above equation sums over the product of the row-specific b_i parameter and the molecule-specific ϕ_{ij}

angle between the geometric dipole of the water molecule and the confinement plane. With these definitions, the $b_i\phi_{ij}$ terms in the summation will have the same sign if the rows of water molecules have alternating dipole directions. Therefore, the absolute value of this order parameter $|\sigma|$ should take on large values for the crystal structure of the flat-rhombic phase, and the motion shown in Fig. 6(a) should correspond to a sign change in this σ order parameter. Given the arbitrary choice of positive values for odd i and negative values for even i in the definition of b_i, only relative changes in the sign of σ should be considered, as the absolute sign of σ has no meaning. Furthermore, note that during the concerted motion described above, the value of the σ order parameter will switch its sign very quickly. If the motion of the protons is not correlated, then the values of the ϕ_{ij} values will be uncorrelated with the row index i. This will cause the σ value to take on intermediate values near 0, which is indicative of a loss of proton order.

Supplementary Note IV. Rotational Autocorrelation Functions

For dynamical insights into the rotational motion of water molecules, we estimate the rotational Legendre autocorrelation functions of the O–H bond with $n = 1$, following the procedure described in Ref. [31]. This autocorrelation function will decay to 0 as the initial rotational configuration of the water molecule relaxes to a random orientation. For each displayed temperature in Supplementary Figure 4(a), we estimate the autocorrelation function over a 25 picosecond window, averaged over 5 trajectories. The definite integral of this function, from 0 to ∞, gives the rotational relaxation time.

Supplementary Figure 4(a) clearly shows two different types of rotational motion in the flat-rhombic phase. As we mentioned in the discussion surrounding Fig. 3 of the main text, water molecules in low-temperature conditions remain localized in their molecular orientations up to the simulation time considered in this work. At low temperatures such as 160 and 220 K, this behaviour leads to a sharp initial decay in the rotational Legendre autocorrelation plots in Supplementary Figure 4(a). This sharp decay is due to thermal fluctuations within the (θ, ϕ) free energy minimum. As a result, these autocorrelation functions exhibit a nearly infinite rotational relaxation time, since the water molecules are stuck in the free energy minimum that they begin in. The diverging relaxation time is an artefact of the absence of molecular rotation sampling within the timescales of our simulations. At intermediate temperatures such as 280 K and 360 K, we observe finite rotational relaxation times, consistent with the (θ, ϕ) exploration of water molecules in Fig. 3 of the main text. We also note a good fit of the rotational relaxation times between 280 K and 360 K to an Arrhenius

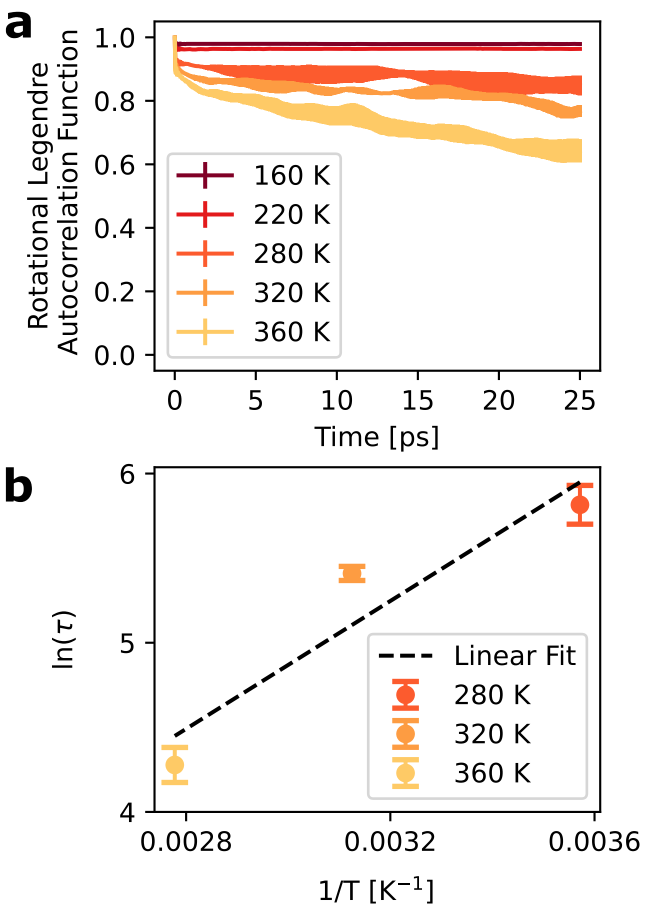

Supplementary Figure 4. | **Rotational Legendre Autocorrelation Functions.** a) The rotational Legendre autocorrelation functions of water molecules in the flat-rhombic phase at various temperatures. These are computed as described in Ref. [31] with $n = 1$. These are averaged over 5 different trajectories. The width of each line is the error, as computed by the standard error of the mean across the trajectories. b) The temperature dependence of the rotational relaxation time τ, fit to an Arrhenius form (dashed black line). We estimate τ as the integral of the above autocorrelation functions, and we only consider temperatures where the concerted motion is observed. The error bars for $\ln(\tau)$ are computed by propagating the errors from the top panel.

equation, suggesting a common activation barrier dictating the rotational motion. We do not include the temperatures 160 and 220 K in the fit, as at these temperatures we do not sample the relevant relaxation event, implying an ∞ relaxation time. We do not account for quantum nuclear motion as they are expected to only make a quantitative difference to this trend [32, 33]. Specifically in the context of the flat-rhombic phase, the correspondence between Fig. 3 of the main text and Supplementary Figure 4, and the quantitative increase in the exploration of (θ, ϕ) orientations due to quantum nuclear effects (see Supplementary Figure 7) implies a systematic increase the relaxation time.

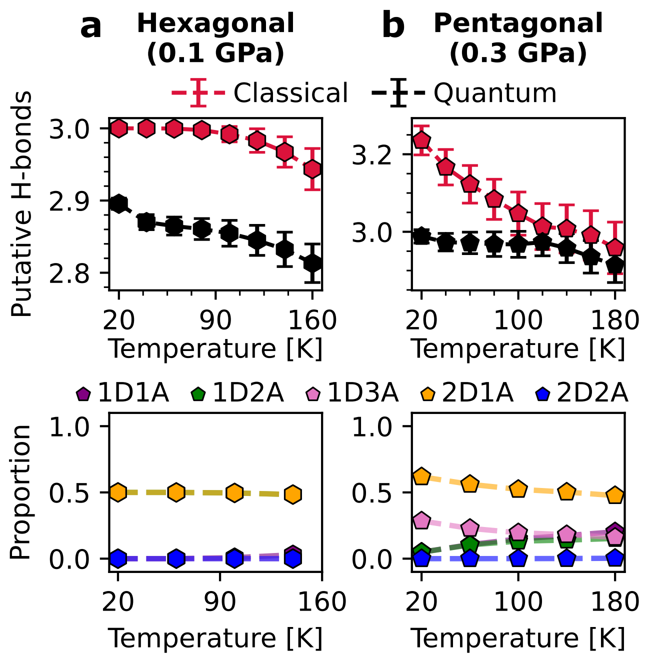

Supplementary Figure 5. | **Hydrogen bonding in the hexagonal and pentagonal phase.** a) Temperature dependence of the number of geometric/putative hydrogen bonds in the hexagonal and pentagonal nanoconfined ice phases. b) The proportion of water molecules occupying each $NDMA$ state for the hexagonal and pentagonal ice phases. The error bars for the top panels show the standard error of the mean, as computed by block averaging over 10 blocks.

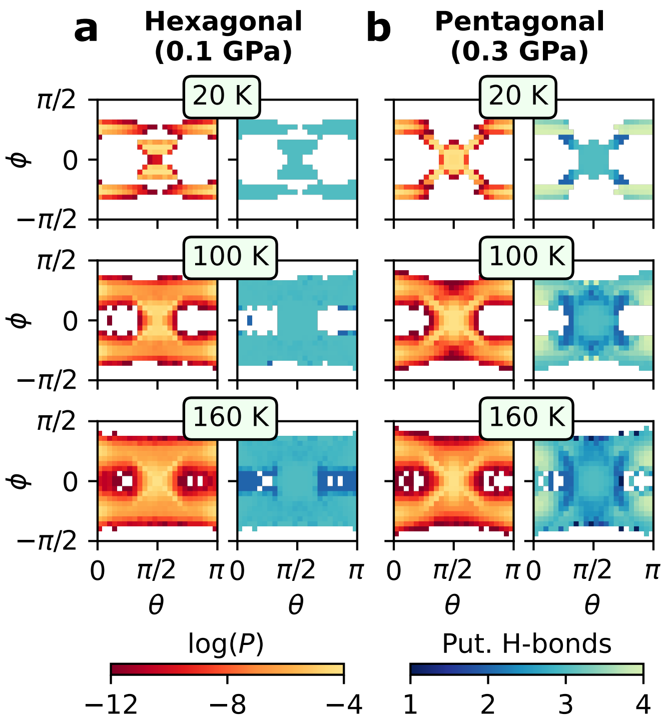

Supplementary Figure 6. | **Molecular orientations in the hexagonal and pentagonal phases.** a) The log-probability and number of hydrogen bonds in (θ, ϕ)-space for the hexagonal phase. b) The same plots for the pentagonal phase.

Supplementary Note V. Hydrogen Bonding Characterization of the Hexagonal and Pentagonal Phases

As shown in Supplementary Figure. 5, the hexagonal and pentagonal phases exhibit decreasing geometric hydrogen bond counts with increasing temperature. This trend is expected based on bulk ice's conventional hydrogen bonding behavior. As seen from the $NDMA$ analysis, in the hexagonal phase, the decline in the number of hydrogen bonds with temperature is due to a slight reduction in the population of the 1D2A / 2D1A states forming 3 hydrogen bonds per molecule and a simultaneous increase in 1D1A states forming 2 hydrogen bonds per molecule. In the pentagonal phase, the decline in the number of hydrogen bonds is due to a reduction in the population of the 2D1A and 1D3A motifs, forming 3 and 4 hydrogen bonds per molecule, respectively, and a simultaneous increase in the population of 1D1A and 1D2A motifs, forming 2 and 3 hydrogen bonds per molecule. Thus, thermal fluctuations disrupt the $NDMA$ states that form the (locally) highest possible number of hydrogen bonds.

In Fig. 3 of the main text, we provide 2D log-probability profiles in ϕ and θ space for molecules in the flat-rhombic phase. We also provide the average number of hydrogen bonds observed in the flat-rhombic phase in ϕ and θ space. Surprisingly, the maxima in the log-probability profiles do not align with the maximum in the number of hydrogen bonds plot. This suggested that the number of hydrogen bonds alone does not predict the stability of a nanoconfined ice configuration. Supplementary Figure 6 shows the same types of plots for the hexagonal and pentagonal phases. These plots serve as a baseline for our expectations from bulk ice, where the Bernal-Fowler ice rules would dictate that bulk ice configurations that satisfy the maximum hydrogen bonding coordination of 4 would be the most stable ordered configurations. The plots in Supplementary Figure 6 for the hexagonal and pentagonal phases exhibit exact alignment between the log-probability maxima and the number of hydrogen bond maxima, which suggests that this hydrogen bonding-oriented picture holds for these phases.

Supplementary Note VI. Impact of Quantum Nuclear Effects on the Flat-Rhombic Phase

Supplementary Figure 7(b) shows the log-probability and average number of hydrogen bonds in (θ, ϕ)-space

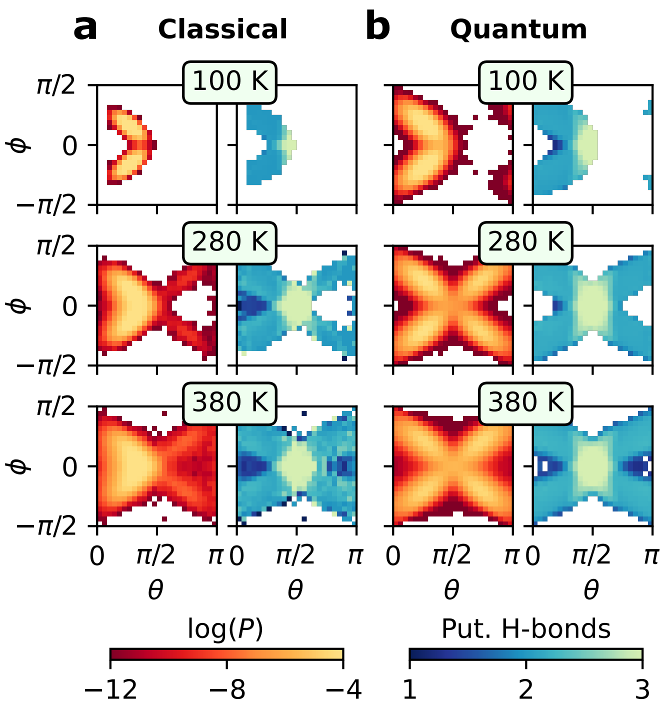

Supplementary Figure 7. | **Nuclear quantum effects and molecular orientations.** a) The classical log-probability profiles and number of hydrogen bond plots in (θ, ϕ)-space. b) The same plots but computed for PIMD simulation trajectories that account for quantum nuclear motion.

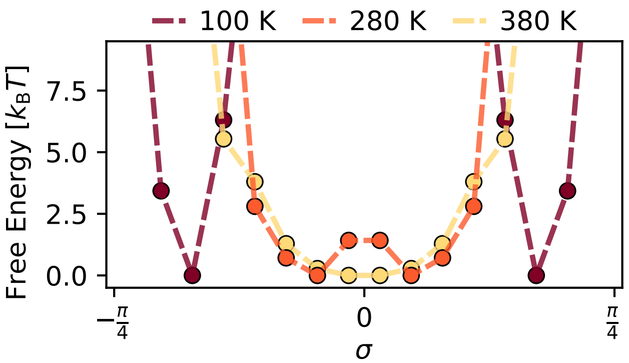

Supplementary Figure 8. | **Nuclear quantum effects and the σ order parameter.** Free energy profiles for the σ order parameter computed from PIMD simulations at various temperatures. This can be directly compared to Fig. 6(c) in the main text.

for the PIMD simulations. Supplementary Figure 7(a) just serves as a convenient replication of the results from Fig. 3(b) of the main text for comparison. The major result, i.e., the fact that the log-probability maxima do not align with the maximum in the number of hydrogen bond plot, holds in the presence of quantum nuclear motion, suggesting that hydrogen bonds are not the primary stabilization interaction in the flat-rhombic phase. However, the PIMD simulations exhibit a broader exploration of (θ, ϕ)-space at all temperatures. In particular, they enhance the in-plane rotational motion of the molecules about its dipole vector.

We also compute the free energy profile along σ in these PIMD simulations in Supplementary Figure 8. Comparing Supplementary Figure 8 to Fig. 6(c) of the main text shows that the classical and PIMD simulations exhibit the same qualitative behavior. However, the free energy barriers for the σ-switching motion are lower due to zero-point fluctuations, and the minima in the free energy profiles move closer to $\sigma = 0$ due to the increased proton disorder, as discussed in the main text.

Supplementary References

[1] Truskett, T. M., Debenedetti, P. G. & Torquato, S. Thermodynamic implications of confinement for a water-like fluid. *The Journal of Chemical Physics* **114**, 2401–2418 (2001). URL https://aip.scitation.org/doi/10.1063/1.1336569.

[2] Zhao, W.-H., Bai, J., Yuan, L.-F., Yang, J. & Zeng, X. C. Ferroelectric hexagonal and rhombic monolayer ice phases. *Chemical Science* **5**, 1757–1764 (2014). URL https://pubs.rsc.org/en/content/articlelanding/2014/sc/c3sc53368a.

[3] Corsetti, F., Matthews, P. & Artacho, E. Structural and configurational properties of nanoconfined monolayer ice from first principles. *Scientific Reports* **6**, 18651 (2016). URL https://www.nature.com/articles/srep18651.

[4] Chen, J., Schusteritsch, G., Pickard, C. J., Salzmann, C. G. & Michaelides, A. Two Dimensional Ice from First Principles: Structures and Phase Transitions. *Physical Review Letters* **116**, 025501 (2016). URL https://link.aps.org/doi/10.1103/PhysRevLett.116.025501.

[5] Chakraborty, S., Kumar, H., Dasgupta, C. & Maiti, P. K. Confined Water: Structure, Dynamics, and Thermodynamics. *Accounts of Chemical Research* **50**, 2139–2146 (2017). URL https://doi.org/10.1021/acs.accounts.6b00617.

[6] Li, S. & Schmidt, B. Replica exchange MD simulations of two-dimensional water in graphene nanocapillaries: rhombic versus square structures, proton ordering, and phase transitions. *Physical Chemistry Chemical Physics* **21**, 17640–17654 (2019). URL https://pubs.rsc.org/en/content/articlelanding/2019/cp/c9cp00849g.

[7] Jiang, J. *et al.* First-Principles Molecular Dynamics Simulations of the Spontaneous Freezing Transition of 2D Water in a Nanoslit. *Journal of the American Chemical Society* **143**, 8177–8183 (2021). URL https://doi.org/10.1021/jacs.1c03243.

[8] Kapil, V. *et al.* The first-principles phase diagram of monolayer nanoconfined water. *Nature* **609**, 512–516 (2022). URL https://www.nature.com/articles/s41586-022-05036-x.

[9] Chen, J., Schusteritsch, G., Pickard, C. J., Salzmann, C. G. & Michaelides, A. Double-layer ice from first principles. *Physical Review B* **95**, 094121 (2017). URL https://link.aps.org/doi/10.1103/PhysRevB.95.094121.

[10] Behler, J. Constructing high-dimensional neural network potentials: A tutorial review. *International Journal of Quantum Chemistry* **115**, 1032–1050 (2015). URL https://onlinelibrary.wiley.com/doi/abs/10.1002/qua.24890.

[11] Zhang, Y. & Yang, W. Comment on “Generalized Gradient Approximation Made Simple”. *Physical Review Letters* **80**, 890–890 (1998). URL https://link.aps.org/doi/10.1103/PhysRevLett.80.890.

[12] Goerigk, L. & Grimme, S. A thorough benchmark of density functional methods for general main group thermochemistry, kinetics, and noncovalent interactions. *Physical Chemistry Chemical Physics* **13**, 6670–6688 (2011). URL https://pubs.rsc.org/en/content/articlelanding/2011/cp/c0cp02984j.

[13] Schran, C., Brezina, K. & Marsalek, O. Committee neural network potentials control generalization errors and enable active learning. *The Journal of Chemical Physics* **153**, 104105 (2020). URL https://aip.scitation.org/doi/10.1063/5.0016004.

[14] Lin, B., Jiang, J., Zeng, X. C. & Li, L. Temperature-pressure phase diagram of confined monolayer water/ice at first-principles accuracy with a machine-learning force field. *Nature Communications* **14**, 4110 (2023). URL https://www.nature.com/articles/s41467-023-39829-z.

[15] Kapil, V., Kovacs, D. P., Csányi, G. & Michaelides, A. First-Principles Spectroscopy of Aqueous Interfaces using Machine-Learned Electronic and Quantum Nuclear Effects. *Faraday Discussions* (2023). URL https://pubs.rsc.org/en/content/articlelanding/2023/fd/d3fd00113j.

[16] Kapil, V., Wilkins, D. M., Lan, J. & Ceriotti, M. Inexpensive modeling of quantum dynamics using path integral generalized Langevin equation thermostats. *The Journal of Chemical Physics* **152**, 124104 (2020). URL https://aip.scitation.org/doi/10.1063/1.5141950.

[17] Shepherd, S., Lan, J., Wilkins, D. M. & Kapil, V. Efficient Quantum Vibrational Spectroscopy of Water with High-Order Path Integrals: From Bulk to Interfaces. *The Journal of Physical Chemistry Letters* 9108–9114 (2021). URL https://doi.org/10.1021/acs.jpclett.1c02574.

[18] Algara-Siller, G. *et al.* Square ice in graphene nanocapillaries. *Nature* **519**, 443–445 (2015). URL https://www.nature.com/articles/nature14295.

[19] Kapil, V. *et al.* i-PI 2.0: A universal force engine for advanced molecular simulations. *Computer Physics Communications* **236**, 214–223 (2019). URL http://www.sciencedirect.com/science/article/pii/S0010465518303436.

[20] Singraber, A., Behler, J. & Dellago, C. Library-Based LAMMPS Implementation of High-Dimensional Neural Network Potentials. *Journal of Chemical Theory and Computation* **15**, 1827–1840 (2019). URL https://doi.org/10.1021/acs.jctc.8b00770.

[21] Thompson, A. P. *et al.* LAMMPS - a flexible simulation tool for particle-based materials modeling at the atomic, meso, and continuum scales. *Computer Physics Communications* **271**, 108171 (2022). URL https://www.sciencedirect.com/science/article/pii/S0010465521002836.

[22] Martyna, G. J., Hughes, A. & Tuckerman, M. E. Molecular dynamics algorithms for path integrals at constant pressure. *The Journal of Chemical Physics* **110**, 3275–3290 (1999). URL https://aip.scitation.org/doi/10.1063/1.478193. Publisher: American Institute of Physics.

[23] Ceriotti, M., Bussi, G. & Parrinello, M. Langevin Equation with Colored Noise for Constant-Temperature Molecular Dynamics Simulations. *Physical Review Letters* **102**, 020601 (2009). URL https://link.aps.org/doi/10.1103/PhysRevLett.102.020601.

[24] Bussi, G., Donadio, D. & Parrinello, M. Canonical sampling through velocity rescaling. *The Journal of Chemical Physics* **126**, 014101 (2007). URL https://aip.scitation.org/doi/10.1063/1.2408420.

[25] Leimkuhler, B. & Matthews, C. Robust and efficient configurational molecular sampling via Langevin dynamics. *The Journal of Chemical Physics* **138**, 174102 (2013). URL https://doi.org/10.1063/1.4802990.

[26] Kapil, V. *et al.* Modeling the Structural and Thermal Properties of Loaded Metal–Organic Frameworks.

An Interplay of Quantum and Anharmonic Fluctuations. *Journal of Chemical Theory and Computation* **15**, 3237–3249 (2019). URL https://doi.org/10.1021/acs.jctc.8b01297.

[27] Chen, J., Zen, A., Brandenburg, J. G., Alfè, D. & Michaelides, A. Evidence for stable square ice from quantum Monte Carlo. *Physical Review B* **94**, 220102 (2016). URL https://link.aps.org/doi/10.1103/PhysRevB.94.220102.

[28] Ceriotti, M. & Manolopoulos, D. E. Efficient First-Principles Calculation of the Quantum Kinetic Energy and Momentum Distribution of Nuclei. *Physical Review Letters* **109**, 100604 (2012). URL https://link.aps.org/doi/10.1103/PhysRevLett.109.100604.

[29] Luzar, A. & Chandler, D. Hydrogen-bond kinetics in liquid water. *Nature* **379**, 55–57 (1996). URL https://www.nature.com/articles/379055a0.

[30] Schienbein, P. & Marx, D. Supercritical Water is not Hydrogen Bonded. *Angewandte Chemie International Edition* **59**, 18578–18585 (2020). URL https://onlinelibrary.wiley.com/doi/abs/10.1002/anie.202009640.

[31] Wilkins, D. M., Manolopoulos, D. E., Pipolo, S., Laage, D. & Hynes, J. T. Nuclear quantum effects in water reorientation and hydrogen-bond dynamics. *The Journal of Physical Chemistry Letters* **8**, 2602–2607 (2017).

[32] Wilkins, D. M. *et al.* Accurate molecular polarizabilities with coupled cluster theory and machine learning. *Proceedings of the National Academy of Sciences* **116**, 3401–3406 (2019). URL https://www.pnas.org/content/116/9/3401.

[33] Habershon, S., Markland, T. E. & Manolopoulos, D. E. Competing quantum effects in the dynamics of a flexible water model. *The Journal of Chemical Physics* **131**, 024501 (2009). URL https://aip.scitation.org/doi/full/10.1063/1.3167790.
