# Supplementary material for: Quasi-one-dimensional hydrogen bonding in nanoconfined ice
Source: Nat Commun. 2024 Aug 24;15:7301. doi: 10.1038/s41467-024-51124-z (PMC11344787; doi:10.1038/s41467-024-51124-z)
Supplement: Supplementary file 3 — Description of Additional Supplementary Files [file 41467_2024_51124_MOESM3_ESM.pdf]

Description of Additional Supplementary Files:

Supplementary Movie 1: A video of the hydrogen bonded switching behavior discussed in the main text. The video shows the row-dependent dipoles switching during the motion, as well as the hydrogen bond network rearranging.
